# Microenvironment-Dependent Gradient of CTL Exhaustion in the AE17sOVA Murine Mesothelioma Tumor Model

**DOI:** 10.3389/fimmu.2019.03074

**Published:** 2020-01-10

**Authors:** Jennifer L. Hope, Panagiota I. Spantidea, Caoimhe H. Kiernan, Christopher J. Stairiker, Laurine C. Rijsbergen, Marjan van Meurs, Inge Brouwers-Haspels, Yvonne M. Mueller, Delia J. Nelson, Linda M. Bradley, Joachim G. J. V. Aerts, Peter D. Katsikis

**Affiliations:** ^1^Department of Immunology, Erasmus MC University Medical Center, Rotterdam, Netherlands; ^2^Cancer Immunology and Tumor Microenvironment Program, Sanford Burnham Prebys Medical Discovery Institute, La Jolla, CA, United States; ^3^Immunology and Cancer Group, School of Biomedical Sciences, Curtin University, Perth, WA, Australia; ^4^Department of Pulmonary Medicine, Erasmus MC University Medical Center, Rotterdam, Netherlands

**Keywords:** cancer, CD8+ T cells, T cell exhaustion, tumors, peripheral lymphoid organs

## Abstract

The immune system, and in particular, cytotoxic CD8^+^ T cells (CTLs), plays a vital part in the prevention and elimination of tumors. In many patients, however, CTL-mediated tumor killing ultimately fails in the clearance of cancer cells resulting in disease progression, in large part due to the progression of effector CTL into exhausted CTL. While there have been major breakthroughs in the development of CTL-mediated “reinvigoration”-driven immunotherapies such as checkpoint blockade therapy, there remains a need to better understand the drivers behind the development of T cell exhaustion. Our study highlights the unique differences in T cell exhaustion development in tumor-specific CTL which arises over time in a mouse model of mesothelioma. Importantly, we also show that peripheral tumor-specific T cells have a unique expression profile compared to exhausted tumor-infiltrating CTL at a late-stage of tumor progression in mice. Together, these data suggest that greater emphasis should be placed on understanding contributions of individual microenvironments in the development of T cell exhaustion.

## Introduction

With more than 1.5 million new cancer diagnoses annually in the US alone and increased numbers worldwide, cancer remains a major public health concern with high incidences of morbidity and mortality. The immune system plays a vital part in preventing and eliminating tumor cells. In particular, cytotoxic CD8^+^ T cells (CTL) are well-recognized for their role in surveillance and killing of tumor cells. In many patients, however, CTL-mediated tumor killing ultimately fails in the clearance of cancer cells resulting in disease progression. During chronic viral infection, such as human immunodeficiency virus (HIV) infection or hepatitis C virus (HCV) infection in humans or some strains of lymphocytic choriomeningitis virus (LCMV) in mice, virus-specific CTL develop an “exhausted” phenotype. CTL exhaustion is a cell-intrinsic alteration resulting in decreased functionality and effector capabilities as well as depressed proliferative capacity and increased apoptosis of the cell. This exhaustion has been shown to be induced by chronic antigen stimulation and is primarily mediated by T cell receptor (TCR) stimulation (1, 2). A hallmark of CTL exhaustion is the progressive up-regulation of a selection of surface markers and receptors known collectively as inhibitory receptors (such as CTLA-4, PD-1, Lag3, 2B4, and CD160) (3). Simultaneously, CTL begin to lose effector functions including the ability to proliferate (and to produce IL-2), cytotoxic capacity, and the production of the pro-inflammatory cytokines IFNγ, and TNFα (3). Ultimately, the exhausted cell becomes pro-apoptotic and is prone to activation-induced cell death upon TCR engagement or Fas-mediated apoptosis (4, 5).

Earlier studies have demonstrated that, similar to chronic infection, tumor-specific CTL are functionally exhausted and can be re-invigorated by immune checkpoint blockade. Recently, the inhibitory effects of CTLA-4 and PD-1 have become major targets in cancer immunotherapies with the development of checkpoint blockade therapeutics such as anti-CTLA-4 and anti-PD-1 antibodies (6–9). These blocking antibodies prevent signaling through the inhibitory receptors, thus enhancing T cell function *in vivo* (10). The combined use of anti-CTLA-4 and anti-PD-1 blockade in patients with melanoma cancer has now become a first-line treatment after clinical trials. This therapy has demonstrated the potential efficacy and remarkable reduction of tumor burden in some late-stage melanoma patients (11). Indeed, the major finding that targeting the CTLA-4 pathway via antibody blockade can enhance anti-tumor responses was first demonstrated in a preclinical mouse model (12), highlighting the relevance and usefulness of murine cancer model systems.

Despite these major advances and breakthroughs however, there remains a great need to better understand the mechanisms by which the immune system and CTL fail in the context of solid tumors (13), as not all patients respond to the current antibody blockade therapies (6, 9, 11). We therefore sought to characterize the development of T cell exhaustion in a murine mesothelioma model expressing ovalbumin, AE17sOVA, which exhibits histological and morphological similarities to human mesothelioma tumors (14, 15). In this model, we observed that naïve OT-I CD8^+^ T cells, transgenic CD8^+^ T cells that recognize the SIINFEKL peptide from OVA, adoptively transferred concurrently with tumor cells differentiate into effector CTL by day 15 and developed characteristics of T cell exhaustion by the late end-point day 22. We also observed that the level of exhaustion was site-specific, exhibiting a gradient of T cell exhaustion which was highest in intra-tumor tumor-specific CTL and progressively decreased in the draining lymph node and further declined in splenic tumor-specific CTL. Taken together, these findings demonstrate that spatial and temporal determinants impact the degree of exhaustion in tumor-specific CTL in the AE17sOVA mesothelioma mouse model. Understanding such determinants in mesothelioma may instruct the timing of checkpoint inhibition and optimal location from which neo-antigen-specific CTL are derived for adoptive transfer therapies. Such optimization may lead to an improvement in the efficacy of immunotherapies.

## Materials and Methods

### Animals and Infections

For influenza virus infections and AE17sOVA tumor experiments: C57BL/6 Tg(TcraTcrb)1100Mjb/J (OT-I) were backcrossed with B6.SJL-Ptprca Pepcb/BoyJ (CD45.1^+^) mice (both from the Jackson Laboratory) to generate OT-I CD45.1^+^ mice on the C57BL/6J background. C57BL/6J mice were kept under SPF conditions at Erasmus University Medical Center or at Sanford Burnham Prebys Medical Discovery Institute (an AAALAC certified animal facility). This study was carried out in accordance with the recommendations of the Instantie voor Dierenwelzijn (IvD) (protocols were approved by the IvD), and in accordance with the recommendations of the Sanford Burnham Prebys Medical Discovery Institute Institutional Animal Care and Use Committee (IACUC) (protocol number 18-067).

For influenza virus infections: 8–10 week-old female mice received an intravenous injection of 1 × 10^4^ OT-I CD45.1^+^ CD8^+^ T cells from an uninfected OT-I female mouse of 8–10 weeks of age; 3 h later, mice were anesthetized with 2.5% isoflurane gas and were infected intranasally with influenza virus strain A/WSN/33 expressing OVA_(257−264)_(WSN-OVA_(I)_; a gift from D. Topham, University of Rochester Medical Center).

For tumor injections: 8–10 week-old female mice received an intravenous injection of 1 x 10^4^ OT-I CD45.1^+^ CD8^+^ T cells from an uninfected OT-I female mouse 8–10 weeks of age; 3 h later, mice were anesthetized with 2.5% isoflurane gas. The hind flank was shaved, then 5 × 10^5^ AE17sOVA cells, an OVA-expressing murine mesothelioma cell line derived from C57BL/6 mice (14), were injected subcutaneously in 100 μL total volume of sterile 0.9% normal saline.

### Cell Culture

AE17 and AE17sOVA cells were maintained in RPMI 1640 supplemented with 10% FBS, 100 units/mL Penicillin/Streptomycin (ThermoFisher, Waltham, MA), 2 mM L-glutamine (ThermoFisher), 0.05 mM 2-mercaptoethanol (ThermoFisher), and were cultured at 37°C in 5% CO_2_; AE17sOVA media was additionally supplemented with 400 μg/L G418 (ThermoFisher). For all experiments, cells were passaged *in vitro* three times prior to injection into mice. AE17sOVA cells were confirmed to be mycoplasma free and re-checked every 6 months. OVA expression of AE17sOVA cells and OT-I responses were confirmed by the activation of naïve OT-I cells in *in vitro* cultures compared to non-OVA expressing AE17 control cells.

### Flow Cytometry

Single-cell suspensions were generated from spleens and lymph nodes by mechanical disruption and passed through a 40 μM cell strainer (Falcon, San Jose, CA). Lungs and tumors were digested by chopping tissues into 1 mm^3^ sections and incubating sections in tissue-culture treated petri dishes for 2 h in RPMI 1640 containing 3 mg/mL Collagenase A and 0.75 mg/mL DNAse I (both from Worthington Biochemical, Lakewood, NJ). Cells were stained as previously described (16). Briefly, in all stains, cells were pre-treated with anti-CD16/32 (Fc Block; 2.4 G2; BioLegend, San Diego, CA) for 15 min before continuing with surface staining. For surface stains, cells were stained for 20 min on ice. Cells were stained with the following fluorochrome conjugated monoclonal antibodies: CD8a (clone 53–6.7), CD45.1 (clone A20), CD45.2 (clone 104), Thy1.1/CD90.1 (clone HIS51) (all from eBioscience/ThermoFisher, San Diego, CA), CD25 (clone PC61), CD69 (clone H1.2F3), CD44 (clone 1M7), CD62L (clone MEL-14) (all from BD Bioscience, San Jose, CA), KLRG1 (clone 2F1/KLRG1), IL-7R/CD127 (clone A7R34), PD-1 (clone 29F-IAI2) (all from BioLegend, San Diego, CA). Cells were also stained with Cy5.5-labeled Annexin V (BD Biosciences) and APC labeled-tetramers of H-2^b^ major histocompatibility complex class I loaded with OVA_(257−264)_ (prepared in the lab) (17) (e.g., SIINFEKL). After staining, cells were washed 2 times with HBSS containing 3% FBS and 0.02% sodium azide and fixed with 1% formaldehyde. For Annexin V staining, all buffers contained 2.5 mM CaCl_2_. For staining of intracellular cytokines, cells were stimulated with SIINFEKL peptide (Anaspec, Fremont, CA) for 6 h at 37°C, 5% CO_2_ in the presence of GolgiPlug (BD Biosciences) and a fluorochrome-conjugated monoclonal antibody against CD107a (clone ID4B) or isotype control. Cells were surface stained as above including additional CD107a antibody (clone ID4B) or the appropriate isotype control (both Biolegend), then fixed overnight at 4°C with IC Fixation Buffer, washed using Perm/Wash buffer (eBioscience) and stained for intracellular cytokines for 45 min at 4°C. Fluorochrome conjugated anti-IFNγ monoclonal antibody (clone XMG1.2), anti-TNFα monoclonal antibody (clone MP6-XT22) or the appropriate isotype controls (all from eBioscience) were used for intracellular stains. After staining, cells were washed twice with Perm/Wash buffer (eBioscience) and fixed with 1% formaldehyde. For staining of transcription factors, cells were surface stained as above then fixed for 1 h at 4°C with FoxP3 Fixation Buffer, washed using Perm/Wash buffer (eBioscience) and stained for transcription factors for 1 h at 4°C. The following antibodies were used in combination with intracellular flow cytometry: anti-T-bet antibody (clone 4B10, BioLegend), anti-Eomes antibody (clone DAN11MAG, eBioscience), anti-Ki67 antibody (clone 16A8, BioLegend), or the appropriate isotype controls. After staining, cells were washed twice with Perm/Wash buffer (eBioscience) and fixed with 1% formaldehyde. All samples were collected with an LSR-Fortessa (BD Biosciences) and analyzed with FlowJo v10 software (Treestar, Ashland, OR). For some figures, the program SPICE (Simplified Presentation of Incredibly Complex Evaluations) (18) was used to generate bar graphs and pie graphs. In brief, Boolean gating was performed on donor OT-I CD8^+^ T cells for the indicated populations using FlowJo v10 (BD Biosciences). The data were then imported into SPICE to generate the figures shown here.

### tSNE Analysis

In brief, the Unbiased hierarchal t-Distributed Stochastic Neighbor Embedding (tSNE) analysis was performed using the tSNE FlowJo v10 plugin on a single pooled sample of donor cells reflective of an equal number of cells from each mouse and each tissue (tumor, spleen, tumor draining lymph node). The following parameters were assessed: PD-1, 2B4, CD69, T-bet, and Eomes. In detail, on a per experiment basis, CD8^+^CD45.1^+^ donor OT-I cells were concatenated within each tissue, using compensated parameters. Concatenated files from like tissues of individual mice were further concatenated using compensated parameters to form three total samples: one each of spleen, tumor draining lymph node (DLN), and tumor. Each file was down-sampled to 1,220 total events reflective of the greater population. The three down-sampled populations were concatenated into one file, then tSNE was run for 1,000 iterations with the following selected parameters: PD-1, 2B4, CD69, T-bet, and Eomes.

### Statistics

For flow cytometry, the normality of the population distribution was assessed using the Shapiro-Wilk normality test by GraphPad Prism 8. Significant differences between normally distributed populations were assessed using a two-tailed, unpaired *t*-test; significant differences between non-normally distributed populations were assessed using a two-tailed Mann Whitney exact test. The tests performed are denoted in each figure legend and subsequent *p*-values are annotated in the associated figure.

## Results

### OVA-Specific CTL Become Progressively Exhausted in the AE17sOVA Mesothelioma Tumor Model and Lose Proliferative Capacity

**Figure 1:**
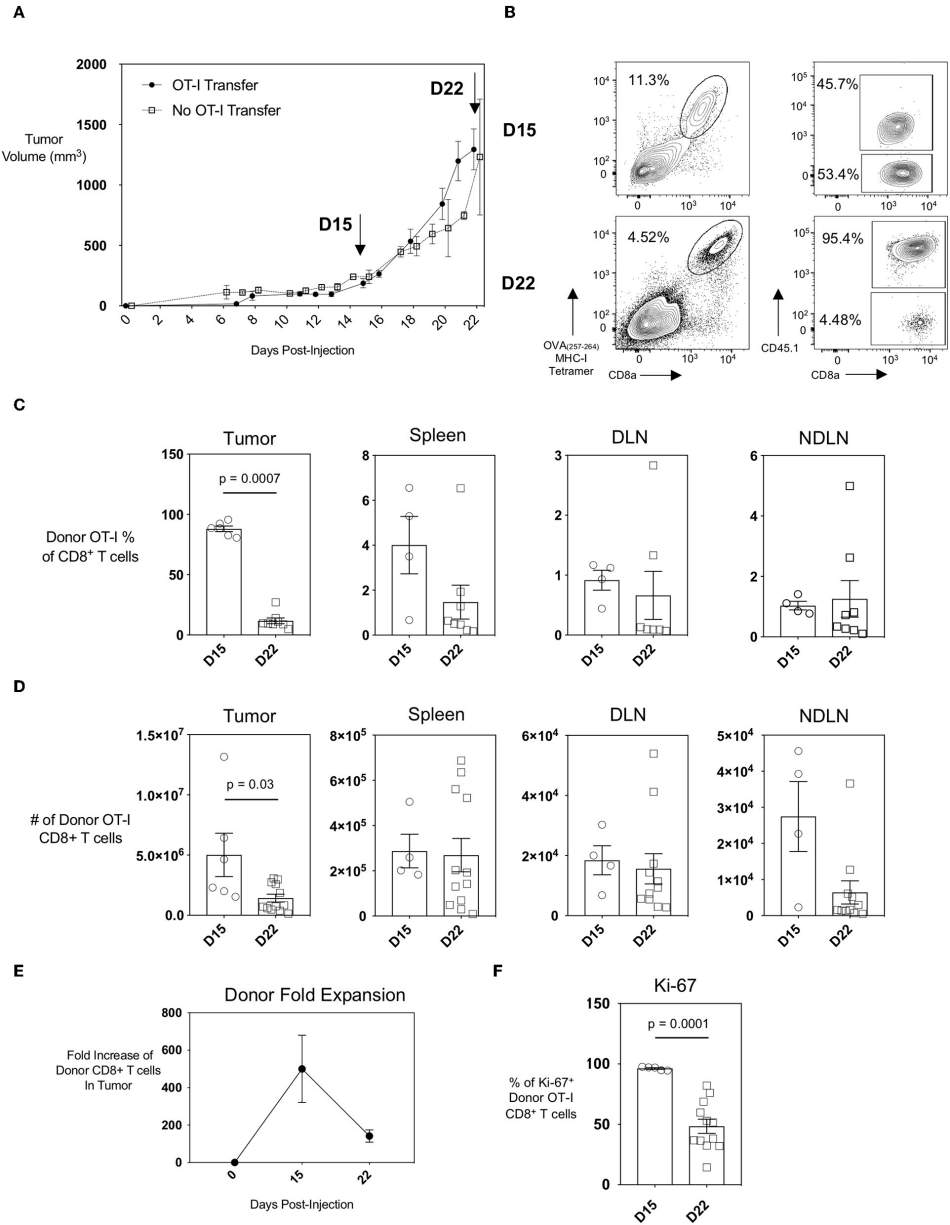
Recruitment and expansion of donor OT-I CD8+ T cells to the tumor site and peripheral lymphoid organs upon injection of AE17sOVA mesothelioma tumor cells. Adult (8–10 weeks) female C57BL/6 mice received an intravenous injection of 1 × 10^4^ naïve OT-I CD45.1^+^ CD8+ T cells followed 3 h later by a subcutaneous injection of 5 × 10^5^ AE17sOVA murine mesothelioma tumor cells in the hind flank. **(A)** Solid, measurable AE17sOVA tumors developed over time, reaching an average of 100 mm^3^ in size at approximately 10 days post-injection. Tumor size was recorded every other day, and tumors, inguinal draining (DLN) and non-draining (NDLN), and spleens were collected at the indicated time points. Data is representative of >3 independent experiments, *n* = 8 mice with OT-I and *n* = 7 mice without OT-I donor cells. **(B)** Representative FACS plots showing donor OT-I CD8^+^ T cells and endogenous tumor-specific CD8^+^ T cells within AE17sOVA tumors of mice 15- and 22-days post-injection of tumor and the frequency of donor vs. endogenous OVA-specific CD8^+^ T cells as determined by staining with MHC class I tetramer with SIINFEKL peptide. Dot plot/bar graph quantifying the individual and average mouse frequencies **(C)** and absolute numbers **(D)** and overall average frequency of donor OT-I tumor-specific CD8+ T cells in the tumors, inguinal lymph nodes, and spleens. **(E)** Dot plot demonstrating the fold-expansion of donor T cells within the tumors from the original 1 × 10^4^ T cell infusion. **(F)** Dot plot/bar graph quantifying the individual mouse and overall average Ki67 expression (MFI, median fluorescence intensity) in intratumoral OT-I CD8^+^ T cells at D22 relative to D15. For experiments B-F, data is representative of at least two experiments, *n* = 4 (spleen, DLN, NDLN) at D15, *n* = 5 (tumor) at D15, *n* = 11–12 mice D22 AE17sOVA tumor injection. The frequencies and absolute cell counts of D15 samples were normally distributed and D22 samples were not normally distributed. Two-tailed exact Mann Whitney tests were used to assess significance and significant data (*p* < 0.05) is annotated within the individual graphs. The Shapiro-Wilk test determined that both D15 and D22 samples were normally distributed for Ki67 expression. A two-tailed unpaired *T*-test was used to assess significance (*p* = 0.0001).

The AE17 mesothelioma cell line was generated by the injection of asbestos fibers intraperitoneally into C57BL/6 mice. To generate the AE17sOVA tumor cell line, AE17 tumor cells were transfected with full-length secretory ovalbumin (OVA) (14, 15). AE17sOVA cells can generate *in vivo* OVA-specific T cell responses (14) and this model is therefore suitable for *in vivo* tracking of both endogenous and adoptively-transferred antigen-specific CD8^+^ T cells. We found that subcutaneous injection of 5 × 10^5^ AE17sOVA into the hind-flank of adult female wild-type mice that had received an injection of 1 × 10^4^ OT-I CD8^+^ T cells 3 h prior resulted in the reproducible growth of vascularized tumors (Figure 1A). This growth curve allowed us to identify two time points of interest: day 15, a time point characteristic of slow but steady tumor growth; and day 22, when tumor growth had increased. At day 15 and day 22 post-injection of both tumor cells and OT-I T cells, the tumors, spleen, and draining and non-draining inguinal lymph nodes (DLN and NDLN, respectively) were collected and analyzed by flow cytometry. The adoptive transfer of 1 × 10^4^ naïve OT-I CD45.1^+^ CD8^+^ T cells followed by hind-flank injection of 5 × 10^5^ AE17sOVA tumor cells into recipient mice resulted in a robust intratumoral donor cell response. Donor cells were identified by the congenic marker CD45.1 and an appreciable endogenous CD8^+^ T cell response was detected by tetramer staining of CD45.1^−^ (host) OVA_(257−264)_-specific CTL (Figure 1B). We additionally observed the recruitment of tumor-specific donor OT-I cells into the inguinal DLN (tumor-bearing side) and NDLN (non-tumor-bearing side) and spleens of mice (Figures 1C,D). We next sought to evaluate if the intratumoral antigen-specific CTL were exhausted, and how early an exhausted CTL phenotype was established within the AE17sOVA tumor model. We observed a substantial expansion and recruitment of donor OT-I T cells into the tumor at day 15 [5.0 × 10^6^ ± 4.1 × 10^6^]. However, this expansion did not persist after day 15 as by day 22 donor OT-I cells decreased in frequency (87.97% of total CD8^+^ T cells at day 15 vs. 11.72% of total CD8^+^ T cells at day 22; *p* < 0.001) and subsequently the total number of donor OT-I T cells compared to the earlier time point decreased also (5.0 × 10^6^ ± 4.1 × 10^6^ on day 15 vs. 1.4 × 10^6^ ± 1.1 × 10^6^ on day 22) (Figure 1D). No significant changes in absolute OT-I CTLs were observed in the spleens, DLN, or NDLN (Figure 1D). Thus, donor OT-I cells within the tumor microenvironment expanded by 100-fold during the first 15 days of slow tumor growth but contracted during the subsequent exponential tumor growth period (Figures 1A,E).

Loss of proliferative capacity is a hallmark of T cell exhaustion, and we reasoned that this may explain the decreased numbers of donor OT-I T cells in the tumors at day 22 post-injection of tumor cells. We therefore assessed the level of Ki-67, a nuclear protein expressed during all stages of the cell cycle (19), in the donor OT-I CD8^+^ T cells as a marker of T cell proliferation. The frequency of donor OT-I T cells expressing Ki-67 was significantly less in day 22 intratumoral donor OT-I T cells compared to day 15 donor T cells [96.26 ± 1.5 % day 15 vs. 48.44 ± 20.29 % day 22; *p* < 0.001], indicating that fewer cells were proliferating at the later time point (Figures 1E,F). Therefore, the progressive decrease of donor OT-I cells in the tumors most likely reflects the reduced ability of these donor cells to proliferate within the tumor by day 22.

### Progressive Exhaustion With Loss of Polyfunctional Cytokine Producing Capability in Donor OT-I Cells by Day 22 Post-tumor Injection

As an example of a highly efficient effector CTL response, we chose to use anti-viral CTL during the primary response to influenza virus for comparison with tumor-specific CTL. Influenza-specific effector CTL are capable of clearing virally-infected cells *in vivo* (20) and thus served as a basis for comparison with the progressive functional exhaustion of anti-tumor CTL. In both tumor and influenza virus models, we utilized adoptive transfers of OT-I cells, thus circumventing possible bias introduced by TCRs of varying affinities being recruited in the respective responses. Effector CTL responding to an acute viral infection such as influenza A virus will upregulate activation markers and be recruited to the site of viral infection such as the lungs. Similarly to influenza virus-specific cells during acute infection, we found that at day 15 tumor-specific (OVA_(257−264)_ MHC-I Tetramer^+^ OT-I) effector CTL that had migrated into the tumor site displayed an activated phenotype including CD44 upregulation and simultaneous downregulation of CD62L, IL-7R (CD127) expression, and upregulation of CD69 (an early marker of T cell activation) (Supplementary Figures 1A–E). In comparison, tumor-specific CD8^+^ T cells in spleens, DLN and NDLN largely maintained IL-7R expression despite CD44 upregulation, and upregulation of CD69 was observed predominantly within the DLN (Supplementary Figures 1F,G). We also observed upregulation of KLRG1 by the OVA-specific CD8^+^ T cells in all tissues tested, and to a greater degree than influenza-specific donor OT-I CD8^+^ T cells (Supplementary Figures 1H,I).

**Figure 2:**
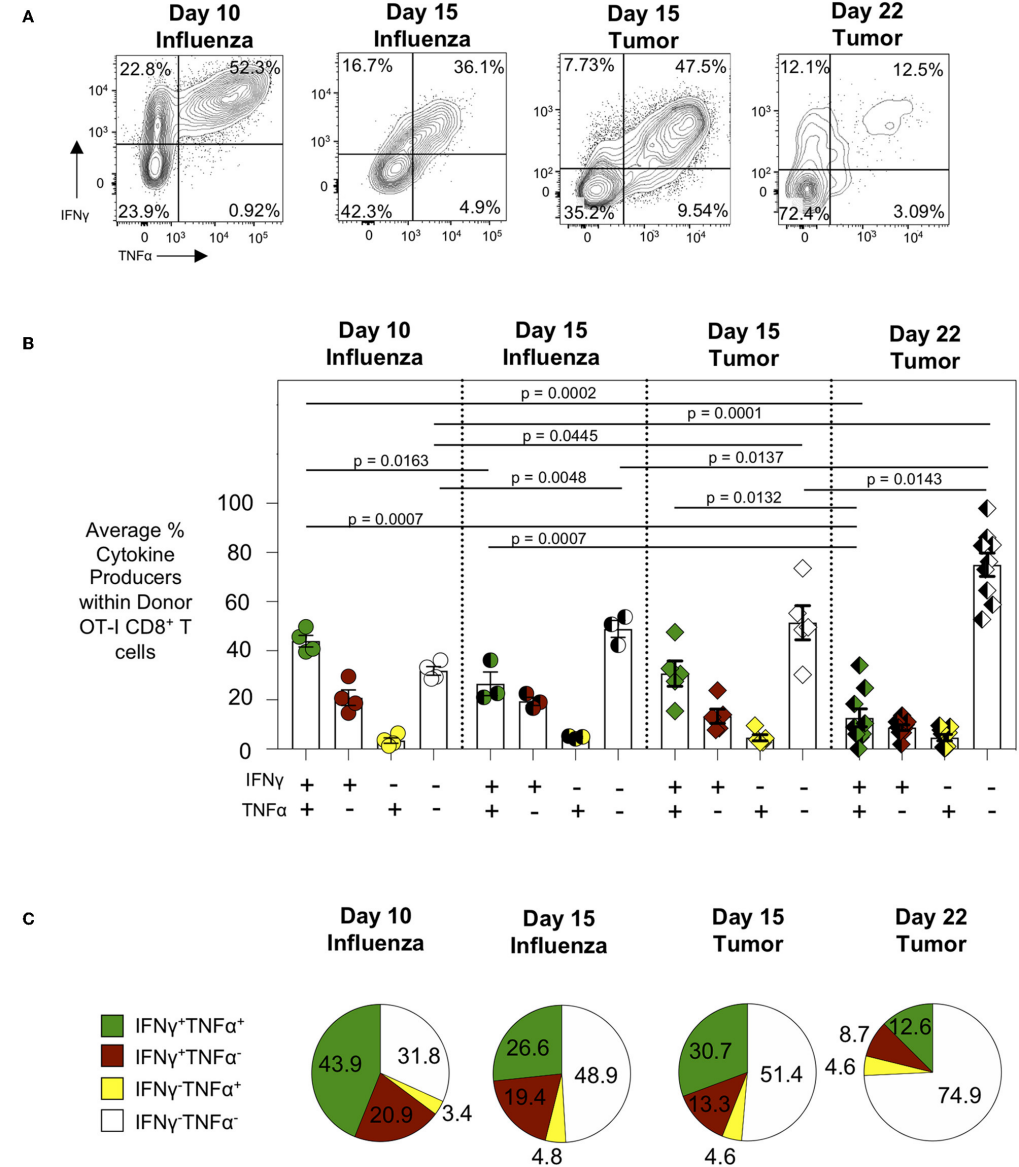
Progressive loss of cytokine production by intratumoral tumor-specific. Intratumoral donor OT-I CD8^+^ T cells were re-stimulated with SIINFEKL peptide (10 uM) for 5 h in the presence of Brefeldin A, followed by intracellular cytokine staining to detect cytokine-producing donor (CD45.1^+^) OT-I CD8^+^ T cells. **(A)** Representative FACS plots showing IFNγ and TNFα cytokine production by donor OT-I CD8^+^ T cells. **(B)** Bar graph comparing IFNγ and TNFα double positive (DP), single positive (SP), or null producing donor OT-I CD8^+^ T cells at day 10 post-infection with WSN-OVA influenza (lungs) and days 15 and 22 post-injection of T cells and tumors (tumors). **(C)** Pie charts comparing DP, SP, or null cytokine producing donor OT-I CD8^+^ T cells from the lungs at day 10 post-infection with WSN-OVA or from the tumors at day 15 or day 22 post-injection of tumor cells. FACS analysis was performed using FlowJo v10, and data was imported into SPICE 6.0 for bar graph and pie chart figure production. For experiments **(A–C)**, data is representative of at least two experiments, *n* = 4 at D10 WSN-OVA influenza virus infection, *n* = 5 at D15, and *n* = 12 mice D22 AE17sOVA tumor injection; data is representative of one experiment, *n* = 3 at D15 WSN-OVA influenza virus infection. All groups except D15 TNFα were normally distributed. When data was normal, a two-tailed unpaired *T* test was used to assess significance; when one sample set was non-normally distributed, a two-tailed exact Mann Whitney test was used to assess significance. Significant data (*p* < 0.05) is annotated within the graph.

We next evaluated the cytokine production of tumor-specific CTL and in particular their polyfunctionality as loss of such polyfunctionality is a hallmark of CTL exhaustion (21). To assess polyfunctionality of tumor-specific CTL, tumor cell suspensions were incubated with SIINFEKL peptide, the cognate antigen recognized by OT-I CD8^+^ T cells (22) and produced by the AE17sOVA tumor cell line (14), for 6 h followed by intracellular staining for flow cytometry to assess the production of the pro-inflammatory cytokines IFNγ and TNFα. While intratumoral donor OT-I T cells 15 days post-injection demonstrated the capacity to produce high levels of IFNγ either alone (13.35 ± 6.43%) or in combination with TNFα (30.72 ± 11.52%) (Figures 2A–C), the frequency of polyfunctional cytokine producing intratumoral donor OT-I T cells 22 days post-injection was significantly reduced (12.65 ± 10.96% day 22 vs. 30.72 ± 11.52% day 15; *p* = 0.0132) (Figures 2A–C). When comparing the frequency of cytokine producing tumor antigen-specific cells to day 10 OT-I from lungs of WSN-OVA influenza virus infected mice, it was clear that while OT-I from day 15 tumors and influenza virus primary response retained the ability to produce cytokines and in particular co-produce multiple cytokines, by day 22 the vast majority of OT-I produced no cytokines and very few cells retained polyfunctional capacity (Figures 2B,C). To ensure that the decreased cytokine production was indeed reflective of functional exhaustion and not a result of normal T cell kinetics or contraction, we similarly evaluated the function of donor OT-I CD8^+^ T cells in the lungs of WSN-OVA influenza virus infected mice 15 days post-infection, during the contraction phase of the T cell response to influenza virus infection (Supplementary Figures 2A–C). While we did observe a decrease in frequency of IFNγ and TNFα co-producing donor OT-I cells in the lungs at day 15 compared to day 10 post-influenza virus infection, there was no significant difference between cytokine production between OT-I from day 15 post-influenza virus infection and day 15 AE17sOVA tumors. Conversely, we observed significantly decreased (26.6 vs. 12.6%, *p* = 0.0007) co-production between OT-I from day 15 post-influenza virus infection and day 22 AE17sOVA tumors (Figures 2B,C). Overall, OT-I cells at day 22 post-injection displayed a more quiescent and less functional phenotype compared to intratumoral OT-I cells from day 15 and this is not simply due to changes occurring during the kinetics of an antigen-specific CTL response.

### Intratumoral Donor OT-I Cells Gradually Acquire and Increase Co-expression of Inhibitory Receptors

**Figure 3:**
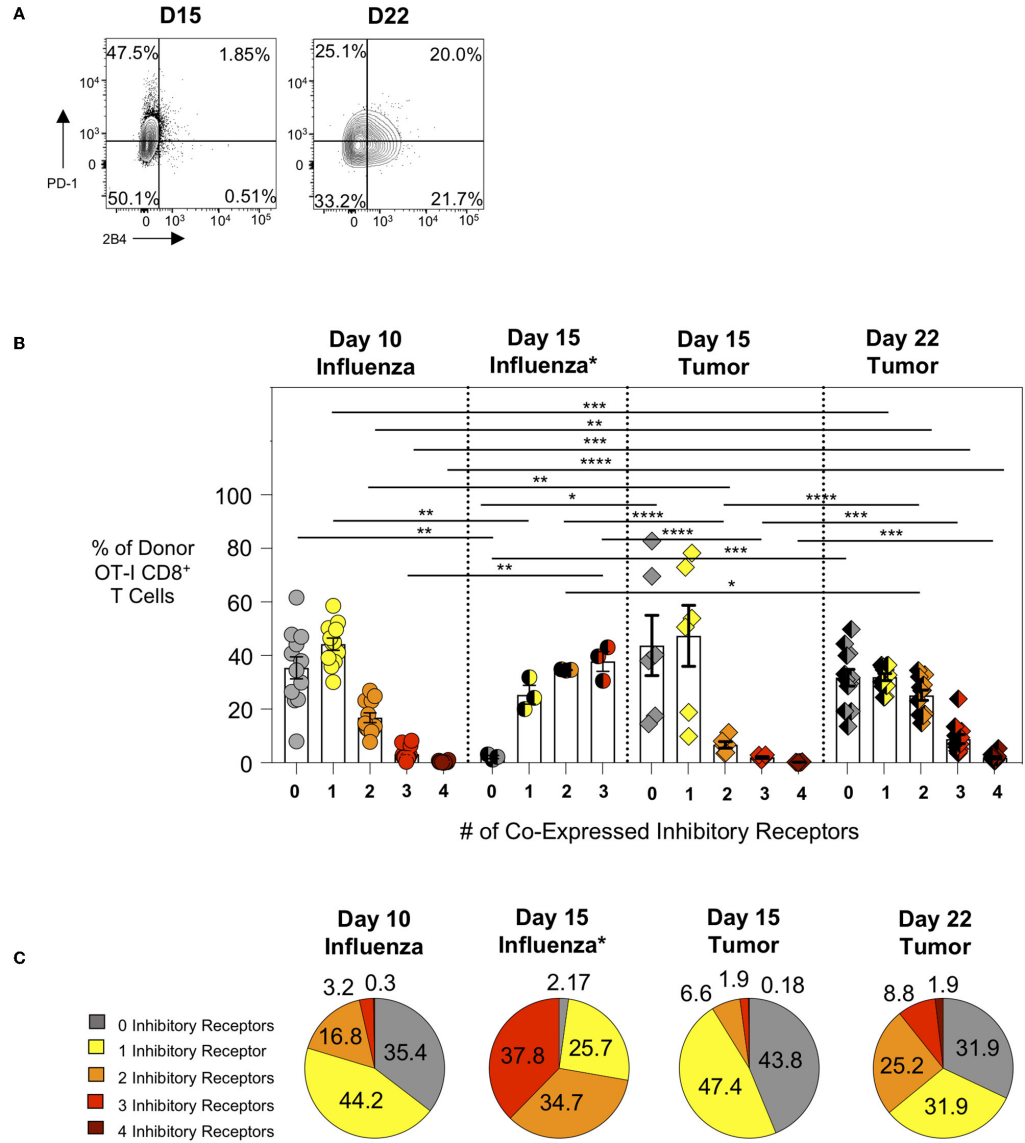
Intratumoral tumor-specific T cells show upregulated inhibitory receptor co-expression at day 22, but not day 15 post-injection. Activation marker/inhibitory receptor (PD-1, Lag3, 2B4, CD160 for influenza D10, tumor D15 and D22; PD-1, Lag3, and TIM-3 for influenza D15) expression was assessed by *ex vivo* flow cytometry analysis of donor OT-I CD8+ T cells from the lungs (WSN-OVA influenza) or tumors at the indicated time points. **(A)** Representative FACS plots showing single or co-expression of PD-1 and 2B4 on donor and endogenous tumor-specific CD8+ T cells from day 15 or day 22 AE17sOVA tumor-bearing mice. **(B)** Bar graph comparing the frequency of donor OT-I CD8^+^ T cells expression of 0, 1, 2, 3, or 4 activation markers/inhibitory receptors (PD-1, Lag3, 2B4, CD160) as determined by Boolean gating. **(C)** Pie charts comparing the overall frequency of inhibitory receptor expression on OT-I CD8^+^ T cells from the lungs at day 10 post-infection with WSN-OVA or from the tumors at day 15 and day 22 post-injection of tumor cells. FACS analysis was performed using FlowJo v10, and data was imported into SPICE 6.0 for bar graph and pie chart figure production. For experiments **(A–C)**, data is representative of at least two experiments, *n* = 4 at D10 WSN-OVA influenza virus infection, *n* = 5 at D15 and *n* = 12 mice D22 AE17sOVA tumor injection; data is representative of one experiment, *n* = 3 at D15 WSN-OVA influenza virus infection. For influenza groups at D10, groups 0, 1, and 2 were normally distributed. For influenza groups at D15, all groups were normally distributed. For AE17sOVA groups at D15, groups 0, 1, 2, and 4 were normally distributed. For AE17sOVA groups at D22, groups 0, 1, and 2 were normally distributed. All others were non-normally distributed. When data was normal, a two-tailed unpaired *T*-test was used to assess significance; when one sample set was non-normally distributed, a two-tailed exact Mann Whitney test was used to assess significance. *p < 0.05; **p < 0.005; ***p = 0.0001; ****p < 0.0001.

We subsequently evaluated the expression of inhibitory receptors on intratumoral CTL as this is another major feature of exhausted CTL. We compared intra-tumor CTL from day 15 and day 22 tumors with effector CTL in lungs 10 days after WSN-OVA influenza virus infection. In both model systems, as expected, PD-1 is upregulated on responding T cells indicating its dual role as both an activation marker and inhibitory receptor (Figure 3A). When comparing the frequency of inhibitory receptor (IR) co-expression between day 10 lungs (influenza) and day 15 tumors (AE17sOVA), we observed an increased frequency in donor OT-I cells expressing 2 IR at day 10 influenza virus infection (Figure 3B). Among intratumoral donor OT-I CD8^+^ T cells at day 22 however, we observed a marked increase in the number of IR co-expression compared to both day 10 influenza virus infection and day 15 AE17sOVA tumors (Figures 3B,C). We also evaluated IR expression on donor OT-I from the lungs of WSN-OVA influenza virus mice at day 15 post-infection; strikingly, we observed a significant increase in IR co-expression compared to both day 10 influenza virus and both day 15 and day 22 tumors (Figures 3B,C, Supplementary Figure 2D). This may be reflective of the donor cells entering the contraction phase of the acute viral infection response and the persistence of antigen in the lungs. This IR expression was not accompanied by functional and proliferative defects and warrants further evaluation in the future. In respect to the intratumoral donor OT-I cells in day 22 tumors, the increased co-expression of the inhibitory receptors PD-1, LAG3, 2B4, and CD160 together with the significantly reduced cytokine production indicates that these cells are more phenotypically and functionally exhausted than their day 15 counterparts (Figures 3B,C).

### The Degree of CTL Exhaustion Development Is Influenced by the Physical Location of CTL

**Figure 4:**
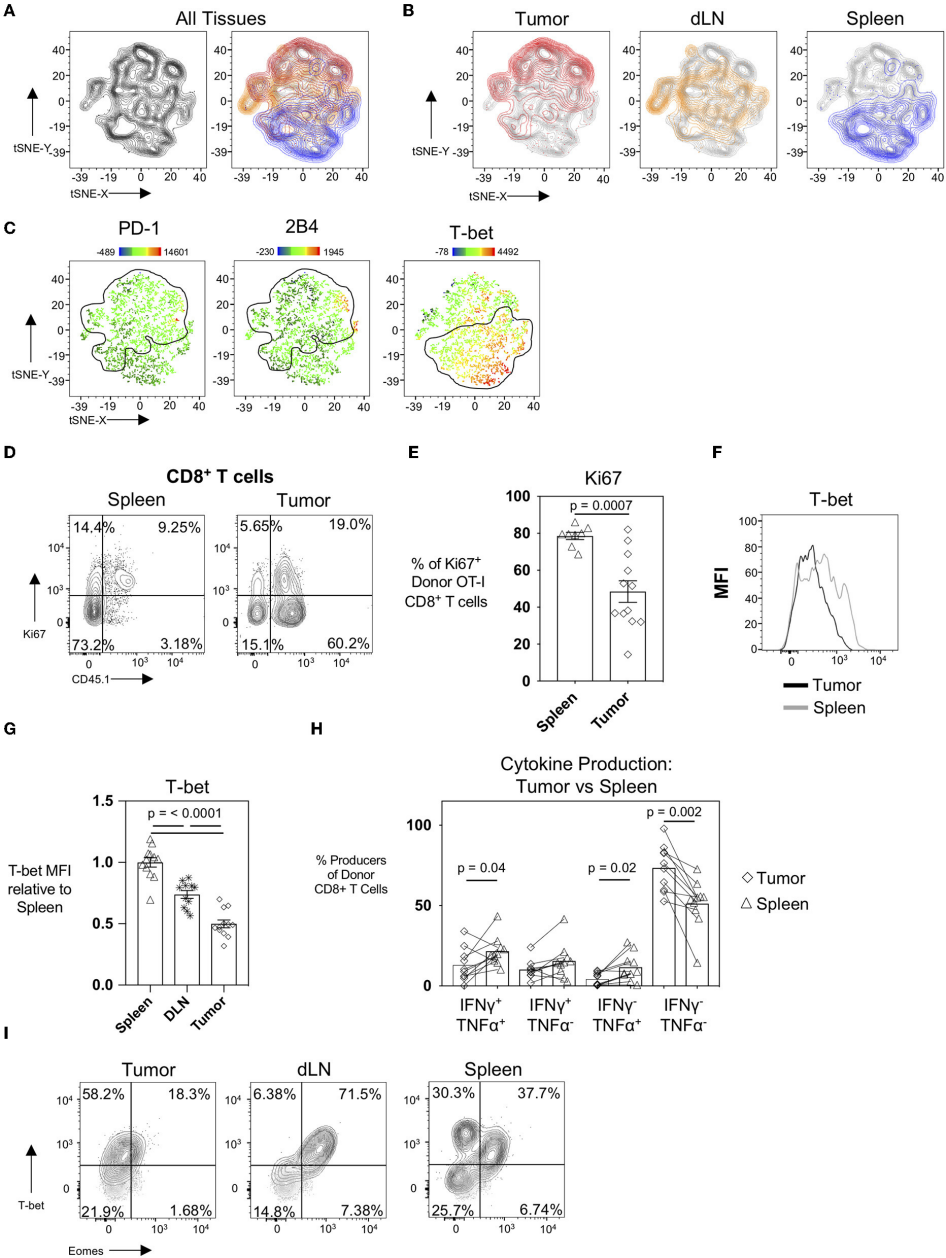
The tissue microenvironment influences the development of exhaustion in CTL. tSNE analysis of donor OT-I CD8^+^ T cells from tumors, tumor DLN, and spleens at day 22 post-injection was performed based on PD-1, 2B4, CD160, T-bet, and Eomes expression using FlowJo v10. **(A)** Contour tSNE FACS plot showing the relatedness of each representative donor cell from the tumors, tumor DLN, and spleens (left) and overlaid with the cells from each tissue of origin (tumor = red, tumor DLN = orange, spleen = blue) (right). **(B)** Overlays of cells from the individual tissues of origin on the overall (gray) contour tSNE FACS plot. **(C)** Median fluorescent intensity gene expression overlays of PD-1, 2B4, and T-bet expression in individual cells. **(D)** Representative FACS plots showing Ki67 expression in CD45.1^+^ donor OT-I CD8+ T cells compared to all CD8^+^ T cells in the spleen (left) and tumor (right) at day 22 post-injection. **(E)** Dot plot/bar graph showing the individual and average frequency of Ki67^+^ donor OT-I CD8^+^ T cells in the respective tissues. **(F)** Representative FACS plot showing T-bet expression in donor OT-I cells in the tumor and spleen at day 22 post-injection. **(G)** Relative T-bet expression in donor OT-I cells in the tumor, spleen, and DLN at day 22 post-injection. **(H)** Dot plot/bar graph comparing the frequency of cytokine-producing donor OT-I T cells from the spleens and tumors of mice at day 22 post-injection. Connecting lines pair matched samples from the same recipient host mouse. **(I)** Representative FACS plots showing T-bet and Eomes expression in CD45.1^+^ donor OT-I CD8^+^ T cells in the respective tissues at day 22 post-injection of AE17sOVA tumor cells. For experiments A-H, data is representative of at least two experiments, *n* = 11–12 mice. All groups were normally distributed except the D22 tumor TNFα SP group. When data was normal, a two-tailed unpaired *T* test was used to assess significance; when one sample set was non-normally distributed, a two-tailed exact Mann Whitney test was used to assess significance. Significant data (*p* < 0.05) is annotated within the graph.

It is well-established that the tumor microenvironment is highly immunosuppressive and modulated by the tumor cells themselves as well as the recruitment of immunosuppressive CD4^+^CD25^+^FoxP3^+^ regulatory T cells (T_regs_), CD11b^+^Gr-1^+^ myeloid-derived suppressor cells (MDSC), and M2-polarized macrophages (23–25). It has also been demonstrated in patients that tumor draining lymph nodes exert immunosuppressive capacity (26) despite also serving as a site for peripheral tumor-specific T cell activation and immune-surveillance (27). To evaluate how different microenvironments and spatial locations could influence the CTL profile, we performed an unbiased hierarchal clustering analysis using the T-distributed Stochastic Neighbor Embedding (t-SNE) machine learning algorithm in FlowJo v10. For this analysis, we compared donor OT-I T cells from the spleens, tumor-draining inguinal lymph nodes, and tumors in mice 22 days post-tumor injection. Individual samples were concatenated, downsampled to equal numbers between tissues, and concatenated again before running tSNE on the expression of the activation and inhibitory receptor markers PD-1, 2B4, CD69, and the transcription factors T-bet (*Tbx21*, a T-box transcription factor) and Eomes (Eomesodermin). The resultant contour tSNE plot (Figure 4A, left) depicts the relative similarities and differences between individual cells from all samples in an unbiased manner. tSNE analysis showed that donor OT-I T cells clustered uniquely with donor OT-I cells isolated from their same tissue of origin (Figure 4A, right). Interestingly, donor OT-I cells from the tumor-draining inguinal lymph nodes displayed an intermediary phenotype between donor cells from the spleen and tumor (Figure 4B). Overlaying PD-1, 2B4, and the transcription factor T-bet expression levels onto the tSNE plots showed expression of PD-1 and 2B4 primarily in the tumor clusters, while T-bet expression was predominately in splenic clusters (Figure 4C). One major difference between the splenic and tumor cells was the percentage of donor cells expressing Ki67. While donor cells within spleens were largely Ki67^+^ (78.58 ± 5.47%), only 48.44 ± 20.29% of donor cells in tumors expressed Ki67 (*p* < 0.001) (Figures 4D,E). This is in contrast to the maintained Ki-67 expression frequency of day 15 donor OT-I cells from WSN-OVA influenza virus in both the lung and spleen (Supplementary Figures 2D,F). Notably, we confirmed that T-bet expression was markedly different in donor OT-I T cells in a tissue-dependent manner (Figures 4F,G). T-bet expression has been associated with effector and effector memory CD8^+^ T cells (28, 29); however, during chronic antigenic stimulation, continued TCR stimulation can result in the loss of T-bet expression in CD8^+^ T cells accompanied by CTL exhaustion (30, 31). Further confirming that this loss of T-bet expression is not simply a result of the kinetics of the CD8^+^ T cell response, we found that influenza virus specific donor OT-I CD8^+^ T cells in the lungs at day 15 post-infection maintained higher levels of T-bet expression compared to donor OT-I cells within the spleens of the same mice (Supplementary Figures 2G,H). Others have described that decreased T-bet and increased expression of another transcription factor, Eomesodermin (Eomes), in T cells is associated with T cell exhaustion, particularly in HIV-specific CD8^+^ T cells (32). We only observed a noticeable shift in T-bet between the day 15 and day 22 intratumoral antigen-specific OT-I T cells, and did not observe upregulation of Eomes in day 22 donor OT-I cells within the tumors compared to the spleen and DLN (Figure 4I).

We additionally compared cytokine production by donor OT-I cells in the spleens to their matched tumors at day 22. Unlike intratumoral donor OT-I CD8^+^ T cells, splenic-derived OT-I CD8^+^ T cells maintained cytokine production (51.18 ± 15.38% cytokine producing donors in spleens vs. 26.54 ± 14.38 cytokine producing donor cells in tumors) at day 22 post-tumor injection; and significantly more polyfunctional donor cells were also found in spleens (21.64 ± 9.149% vs. 12.96 ± 10.38%, *p* = 0.043) (Figure 4H). This is contrary to our findings in day 15 post-influenza virus infection in which the frequency of IFNγ and TNFα co-producing donor OT-I cells was unchanged between the lung and spleen (Supplementary Figure 2B).

The above data taken together, demonstrate a clustering of anti-tumor CTL phenotypes depending on location and a decreasing gradient of anti-tumor CTL exhaustion from the tumor to the draining lymph nodes to the spleen.

## Discussion

Understanding mechanisms driving T cell exhaustion is central to the development and novel application of immunotherapies to different types of cancer. Upon TCR activation, PD-1 is upregulated on effector T cells in response to most stimuli, including in response to acute viral infection such as influenza virus (33). However, continuous TCR stimulation drives maintained and increased expression of PD-1 as well as the co-expression of additional inhibitory receptors, thereafter, serving as a marker of T cell exhaustion. The general mechanisms of PD-1-mediated inhibition of effector T cell responses have been previously described (34), and it is important to note that PD-1, although well-studied, is not the only intrinsic mechanism involved in the development of T cell exhaustion. It is clear that intrinsic molecular pathways including signaling initiated by ligation of other surface molecules such as CD160, TIM-3, and CTLA-4 promote T cell inhibition and play a central role in T cell exhaustion (35–37). Further, the success of antibody-mediated blockade of inhibitory receptors to limit further exhaustion and restore T cell function underscores the need to identify additional targetable mediators and pathways of T cell exhaustion, particularly in the context of cancer (38, 39).

Exhaustion in CD8^+^ T cells can be described as a multi-factorial phenotype based on a combination of surface marker expression (e.g., co-expression of inhibitory receptors), decreased functionality (e.g., decreased pro-inflammatory cytokine production, decreased cytotoxic capability), and decreased proliferative capacity. Together, this results in a CTL population that is incapable of clearing a chronic infection or tumor. Our study clearly demonstrates that the level of exhaustion in CTL is either influenced or determined by the distinct microenvironment where the T cells are isolated. In our studies involving AE17sOVA, we observed that both adoptively transferred OT-I cells and endogenous OVA-specific CTL (data not shown) were characteristically more exhausted when analyzed *ex vivo* from tumors vs. DLNs where an intermediate T cell exhaustion phenotype was observed, and spleens where effector cells did not manifest an exhausted phenotype, as expected. Taken together, the increased inhibitory receptor expression, decreased cytokine production, decreased proliferative capacity, and decreased T-bet expression observed in day 22 post-tumor donor OT-I T cells indicate that tumor-specific T cells in the AE17sOVA mesothelioma tumor model develop characteristic T cell exhaustion. The development of this exhausted T cell (T_EX_) phenotype in the donor OT-I T cells is likely a result of sustained exposure to the tumor microenvironment and prolonged antigen exposure. This is exemplified by the fact that intratumoral donor OT-I T cells assessed at the earlier day 15 timepoint more closely resembled effector influenza-specific donor OT-I T cells from the lungs of mice during the peak of the CTL response to acute influenza virus infection. Our characterization demonstrates that the AE17sOVA mesothelioma tumor model is therefore a suitable model for evaluating the kinetics of T cell exhaustion in the context of cancer, with day 15 intratumoral donor OT-I T cells being “pre-exhausted,” while donor OT-I T cells on day 22 reflect an “exhausted” phenotype for CTL analysis. Our data also suggests that the development of CTL exhaustion in the AE17sOVA murine mesothelioma tumor model has both spatial and temporal requirements. Whereas intratumoral donor OT-I T cells at day 15 post-injection have a phenotype that more closely resembles effector CTL during acute viral infection, day 22 tumor-derived donor OT-I T cells have highly upregulated inhibitory receptor expression, decreased proliferative capacity and cytokine production, and have downregulated T-bet expression levels. By comparison, tumor-specific donor CD8^+^ T cells in the periphery at day 22 demonstrate a gradient of T cell exhaustion based on their physical location. Those T cells within the tumor have the highest measurable level of T cell exhaustion, while T cells within tumor DLN are more exhausted than splenic T cells but less so than intratumoral T cells. In conclusion, these data support the use of the AE17sOVA mesothelioma tumor model as a means of evaluating the effectiveness and timing of anti-tumor therapeutic interventions and enhancing our understanding of the basic requirements for the development of T cell exhaustion in cancer.

The differing levels of CTL exhaustion could be a result of the cellular composition of individual microenvironments, as it is already well-established that the tumor microenvironment can be highly immunosuppressive. Tumor sites are heavily infiltrated by immunosuppressive MDSC, T_regs_, and M2-polarized anti-inflammatory macrophages [see (40)]. Murine mesothelioma induces locally-proliferating IL-10^+^TNF-αCD206^−^CX3CR1^+^ M3 macrophages that can be selectively depleted by chemotherapy or immunotherapy (40). In addition to creating an anti-inflammatory environment, these cells also produce factors that can directly promote tumor growth and vascularization such as matrix metallopeptidase 9 and TGFβ while cytokines produced by the tumor cells (e.g., GM-CSF, IL-6, and VEGF) limit differentiation of myeloid cells and promote MDSC development (41). Production of TGFβ, VEGF, PGE2, and IL-10 by tumor endothelial cells can also act directly on T cells to suppress their function (42). MDSC have also been shown to accumulate in the spleen, blood, and tumor DLN (41). A study from Watanabe et al., demonstrated that reconstitution of lethally irradiated mice with spleen cells from tumor-bearing mice was sufficient to induce T cell suppression within DLNs upon inoculation with tumor cells (41), showing the suppressive capacity of splenic derived MDSCs. Our data clearly shows that by day 22 post-tumor injection, intratumoral donor OT-I T cells express high levels of inhibitory receptors, decreased cytokine production, and reduced proliferative capacity—all of which are indicators of T cell exhaustion. Conversely, donor OT-I T cells within the spleens of AE17sOVA mesothelioma-bearing mice did not display an exhausted phenotype, while T cells in the tumor DLN showed a partially exhausted phenotype when mice were challenged with AE17sOVA mesothelioma tumor cells. While the Watanabe study assessed T cell suppression mediated by splenic MDSCs in new host mice using tumor DLN as a readout, our study addressed overall T cell functionality *ex vivo* from the specific tissue microenvironments.

T_regs_ play a major role in modulating effector T cell responses, and their role in inhibiting effective anti-tumor CTL responses within tumors have been well-described. Largely, a high ratio of T_reg_ to effector T cells within a tumor indicates a worse patient prognosis (43), and in some studies the frequency of T_reg_ within the total CD4^+^ tumor-infiltrating lymphocyte population was found to be >60% (44). While there is evidence of direct T_regs_ suppression of effector T cells via cell-to-cell contact (45), the impact of T_regs_ on the tumor microenvironment and immune cells from the secretion of TGFβ and IL-10 is a major contributor to the immunosuppressive environment of tumors (46). T_reg_-mediated suppression of tumor-specific CTL responses have also been identified within the tumor DLN, such as the identification of TGFβ-secreting T_regs_ in tumor DLN promoting tumor malignancy in breast cancer (47). There is also evidence of early skewing of differentiating CD4^+^ T cells toward induced T_regs_ rather than effector CD4^+^ T cells in the TDLN (48). Further, local effector CTL failure in mesothelioma is not mediated by CD4^+^CD25^+^FoxP3^+^ T_reg_ cells, as while T_regs_ influence CTL responses when tumors are small, other immune suppressor cells (macrophages, in particular) play a major role in inhibiting effector CTL (49). It is therefore clear that these different, spatially-separated microenvironments play a crucial role in regulating and modulating the development of effector T cell responses in cancer; gaining a better understanding of the interplay between the microenvironment and development of CTL exhaustion may also aid the development or application of novel checkpoint blockade therapies.

Another important consideration in the development of T cell exhaustion is antigen abundance. It has previously been demonstrated that during chronic LCMV viral infection, the amount of antigen recognized by T cells early on during infection directly influences their subsequent development into effector or exhausted T cells (2, 50). Using a modified strain of LCMV clone 13 wherein only the gp33 expression level was altered, Utzschneider et al. demonstrated that at 4 weeks post-infection, decreasing the amount of gp33 expression while maintaining all other chronic infection factors limited the development of T cell exhaustion in TCR transgenic P14 T cells (which specifically recognize the gp33 epitope of LCMV). Similar results were observed within the endogenous virus specific CD8^+^ T cells. As many similarities have been observed between exhausted T cells from chronic LCMV infection and cancer models, it is therefore possible that the differences in phenotype observed between the tumor, tumor DLN and spleen are influenced by differing levels of antigen in the environment during early T cell activation. CTL from spleens during LCMV infection are fully exhausted while high viral loads of LCMV can be found in the spleen and many other tissues. It is likely that tumor antigen in AE17sOVA bearing mice, may be found in the spleen as well but at a lower level than the tumor DLN and the tumor itself, and this could then influence the lower level of exhaustion found in the spleen and the intermediate exhaustion phenotype observed in tumor-specific CTL isolated from the tumor DLN. Like B16-OVA, we also believe that AE17sOVA tumors experience the loss of OVA-antigen over time, as we have found lower OVA expression levels in large tumors (unpublished data) and this loss could be reflective of immune evasion in response to SIINFEKL-specific CTLs.

It is apparent that although significant progress has been made in regards to the development of cancer immunotherapies, there remains a need to identify new and enhance existing therapies. There is also a need to define biomarkers that can predict the likelihood that a patient will respond to these therapies, as the current regiment of anti-PD-1 and anti-CTLA-4 treatment can have severe side effects and <50% of patients demonstrate at least a partial response. It is therefore of great use to identify model systems of T cell exhaustion, such as AE17sOVA, for use in studies assessing the effectiveness of combination checkpoint blockade therapy. It is not uncommon for animal studies using tumors that express strong antigens such as OVA to inject mice at a young age, and therefore immunologically immature, or require the use of immune incompetent hosts in order to avoid spontaneous tumor rejection (51). Having a model where immunologically mature mice can be injected with a tumor that carries a model antigen allows for studies of tumor exhaustion that are more relevant to cancer patients. By demonstrating that tissue niches influence the amount of CTL exhaustion, our study also highlights the importance of the environment from which cells are isolated for analysis. For translational applications, analyzing tumor-specific T cell responses from the blood or spleen of tumor-bearing mice may be more relevant when comparing to human PBMCs than T cells isolated from the tumor itself. In conclusion, our study validates the use of the AE17sOVA tumor model system as a mechanism to study CTL exhaustion during cancer in an antigen-specific manner and has yielded new insights into the contribution of different tissue microenvironments in the development of T cell exhaustion *in vivo*.

## Data Availability Statement

The datasets generated for this study are available on request to the corresponding author.

## Ethics Statement

The animal study was reviewed and approved by Instantie voor Dierenwelzijn (IvD), Erasmus University Medical Center; and the Institutional Animal Care and Use Committee, Sanford Burnham Prebys Medical Discovery Institute.

## Author Contributions

JH and PS performed tumor injections, adoptive transfers, and flow cytometry. JH performed influenza infections. CS, LR, MM, YM, and CK performed injections, *in vitro* cultures, flow cytometry, and mouse breeding. JA and LB provided key guidance in experimental design. DN provided the AE17sOVA tumor cell line. JH and PK were responsible for study design, data analysis, and manuscript authorship. All authors discussed the results and commented on the manuscript.

### Conflict of Interest

The authors declare that the research was conducted in the absence of any commercial or financial relationships that could be construed as a potential conflict of interest.
