# Supplementary material for: Microenvironment-Dependent Gradient of CTL Exhaustion in the AE17sOVA Murine Mesothelioma Tumor Model
Source: Front Immunol. 2020 Jan 10;10:3074. doi: 10.3389/fimmu.2019.03074 (PMC6968785; doi:10.3389/fimmu.2019.03074)
Supplement: Supplementary file 1 [file Presentation_1.pdf]

Supplemental Figure 1

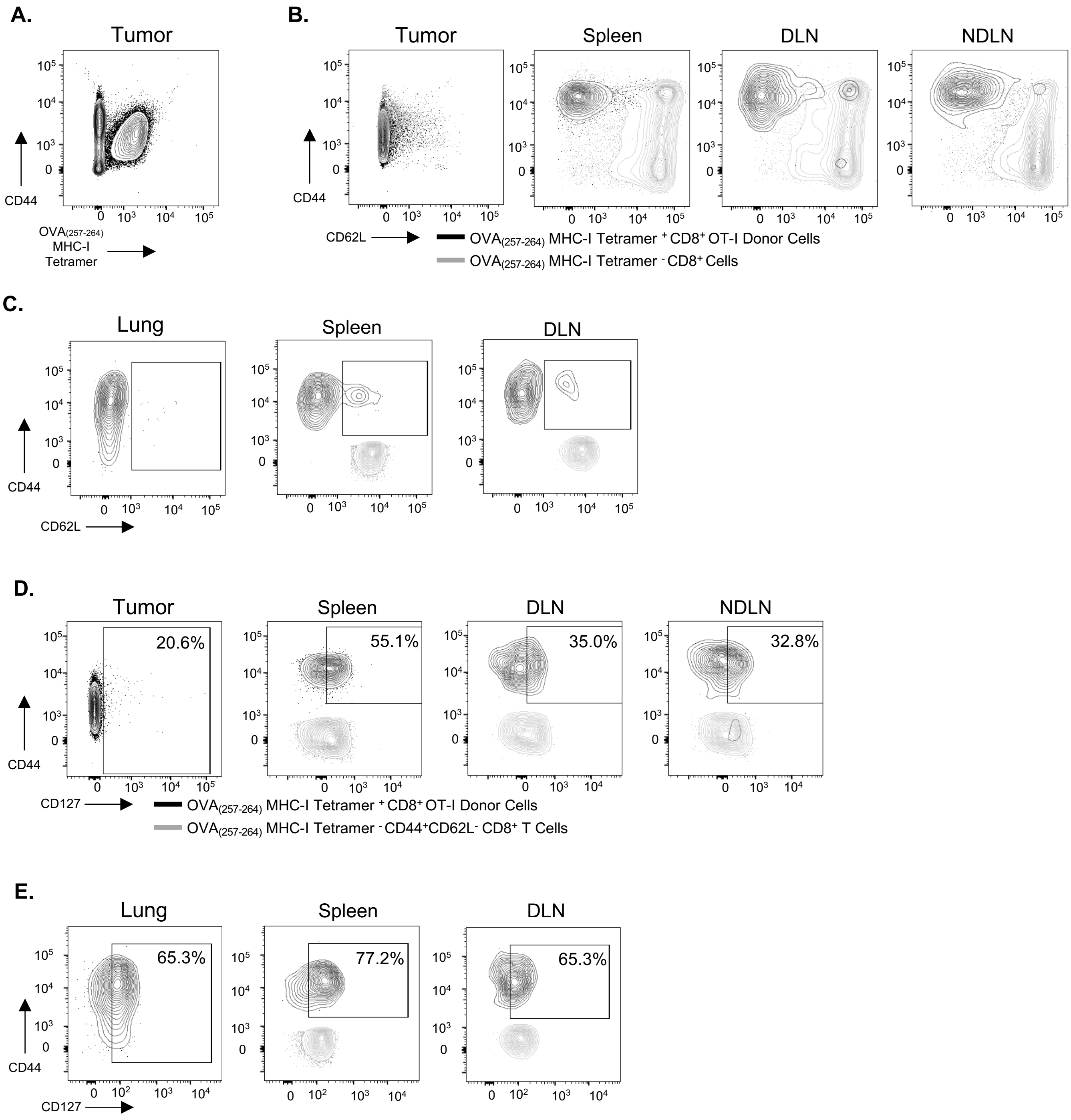

Supplemental Figure 1 cont.

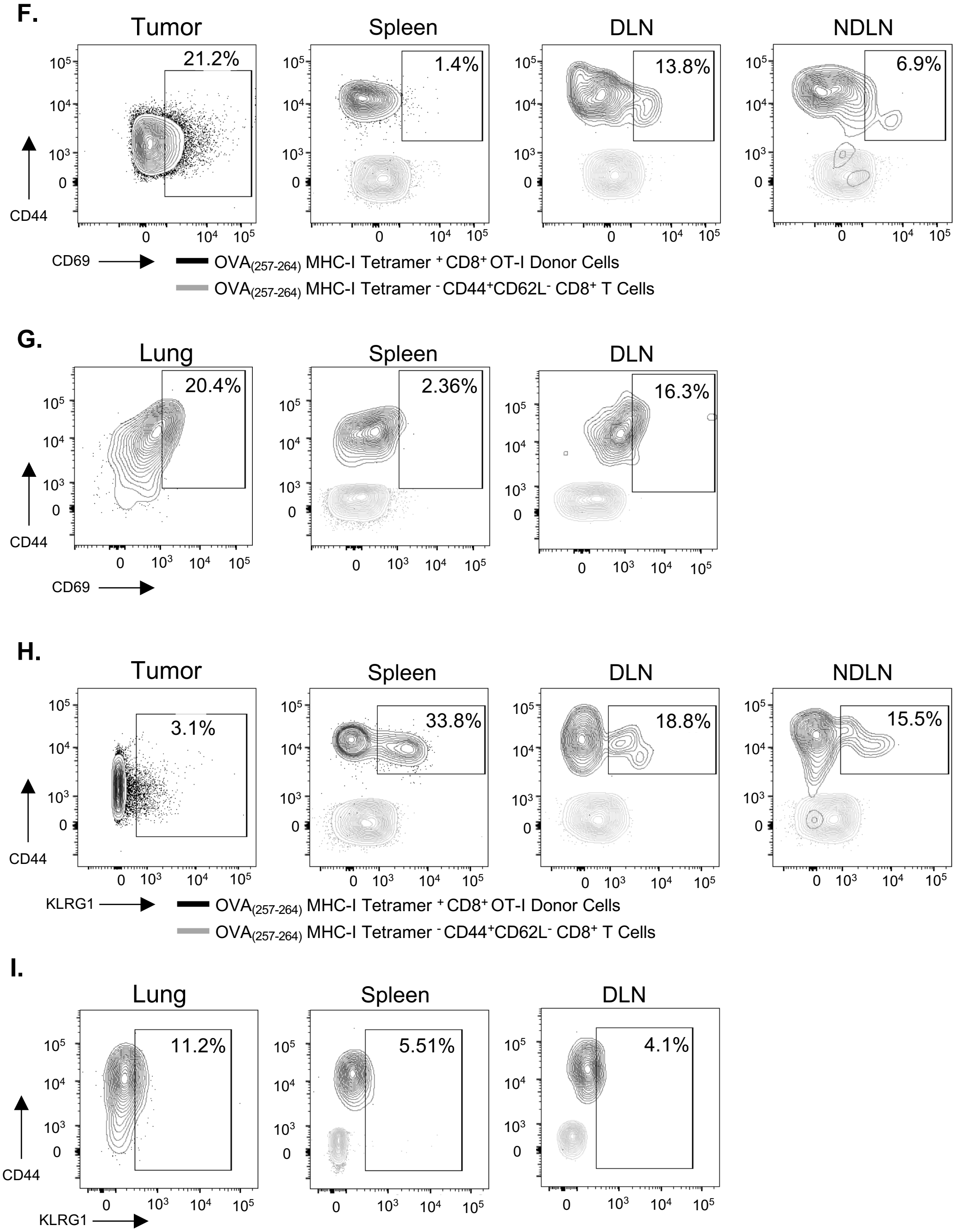

S1. Tumor-specific CTL are more highly activated in the tumor and draining lymph nodes than in the periphery. T cell activation was evaluated for both tumor-specific ($OVA_{(257\text{-}264)}$ MHC-I Tetramer$^+$ OT-I) and non-specific CD8$^+$ T cells in AE17sOVA tumors and the spleens, DLN and NDLN of AE17sOVA tumor-bearing mice 15 days post-injection of donor OT-I CTL and AE17sOVA tumors, and in influenza virus infected mice 10 days post-injection of donor OT-I CTL and infection with WSN-OVA. Representative FACS plots pre-gated on CD8$^+$ T cells are shown comparing the profiles of donor OT-I CTL to non-activated (CD44$^-$CD62L$^+$) CD8$^+$ T cells from the same tissue and mouse. (A) CD44 expression of $OVA_{(257\text{-}264)}$ MHC-I Tetramer$^+$ OT-I donor cells within AE17sOVA tumors. Evaluation of activation status of donor OT-I CTL (black) and non-activated CD8$^+$ T cells (gray) in the indicated tissues from either AE17sOVA tumor-bearing or WSN-OVA influenza virus infected animals. Representative FACS plots comparing the expression of CD44 versus CD62L (B, AE17sOVA; C, WSN-OVA), CD127/IL-7R (D, AE17sOVA; E, WSN-OVA), CD69 (F, AE17sOVA; G, WSN-OVA), or KLRG-1 (H, AE17sOVA; I, WSN-OVA). Frequencies are of the donor OT-I population. Representative of at least 5 mice per group from at least two independent experiments.

Supplemental Figure 2

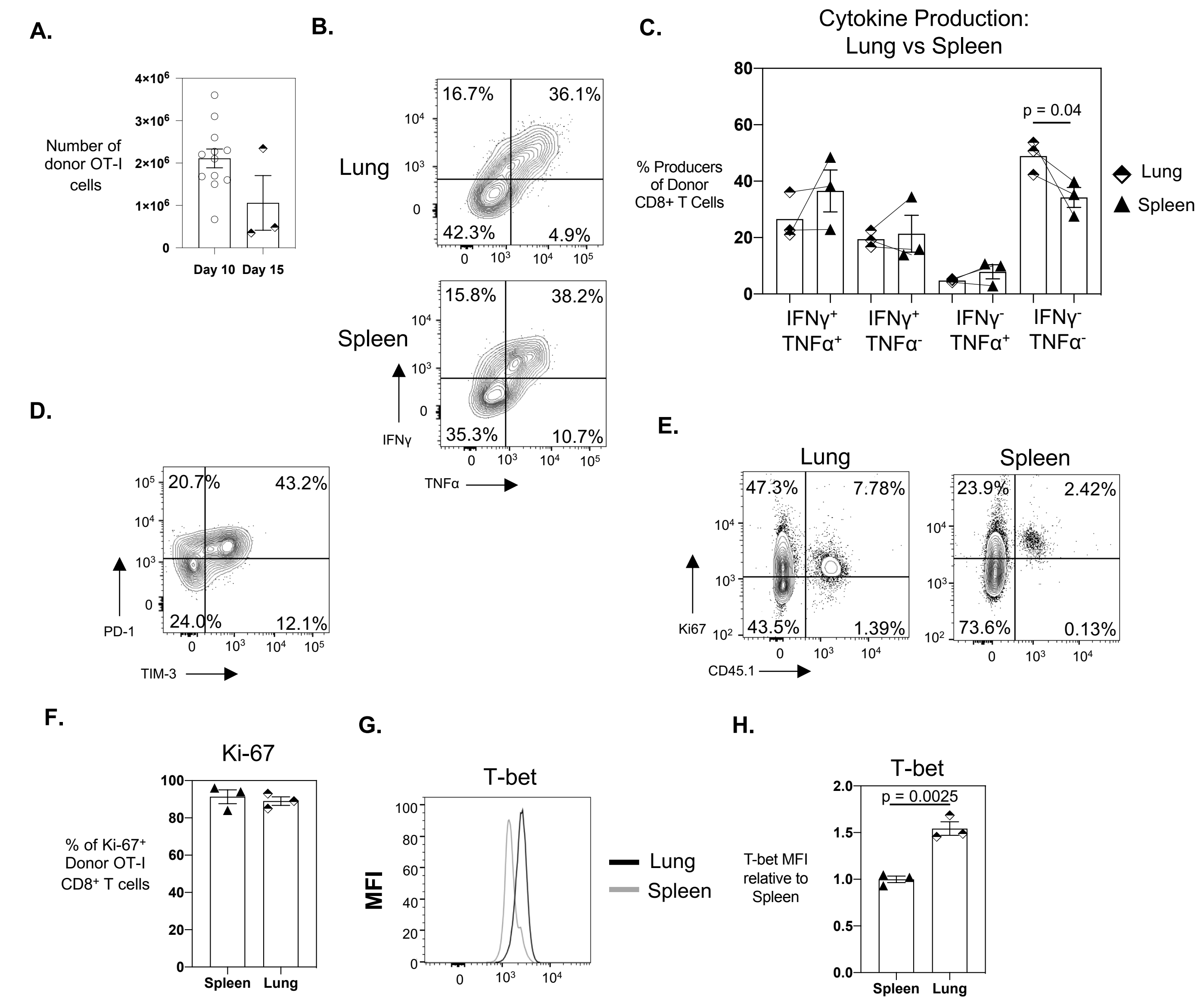

S2. Characterization of WSN-OVA influenza virus-specific donor OT-I CTL 15 days post-infection. (A) Absolute numbers of donor OT-I CD8⁺ T cells in the lungs of WSN-OVA influenza virus infected mice at the indicated time points. **(B)** Representative FACS plots showing IFNγ and TNFα cytokine production by donor OT-I CD8⁺ T cells. Lung and splenic donor OT-I CD8⁺ T cells were re-stimulated with SIINFEKL peptide (10 µM) for 5 hours in the presence of Brefeldin A, followed by intracellular cytokine staining to detect cytokine-producing donor (CD45.1⁺) OT-I CD8⁺ T cells. **(E)** Dot plot/bar graph comparing the frequency of cytokine-producing donor OT-I T cells from the spleens and lungs of mice at day 15 post-infection. Connecting lines pair matched samples from the same recipient host mouse. Data is representative of one experiment, n = 3 mice. All groups were normally distributed, and a two-tailed unpaired T test was used to assess significance. **(D)** Representative FACS plot of PD-1 and TIM-3 co-expression on donor OT-I CD8⁺ T cells in the lungs of mice at day 15 post-infection. Representative of n=3 mice. **(E)** Representative FACS plot of Ki-67 expression in donor OT-I (CD45.1⁺) CD8⁺ T cells in the lung (left) and spleen (right) of WSN-OVA influenza virus infected mice 15 days post-infection. **(F)** Dot plot/bar graph quantifying the individual mouse and overall frequency of Ki-67 expression in OT-I CD8+ T cells. Data is representative of one experiment, n = 3 mice. **(G)** Representative FACS plot showing T-bet expression in donor OT-I cells in the lung and spleen of mice at day 15 post-infection. **(H)** Relative T-bet expression in donor OT-I cells in the spleen and lung of mice at day 15 post-infection. Data is representative of one experiment, n = 3 mice. All groups were normally distributed, and a two-tailed unpaired T test was used to assess significance.
